# The Complement System of Agnathans

**DOI:** 10.3389/fimmu.2018.01405

**Published:** 2018-06-18

**Authors:** Misao Matsushita

**Affiliations:** Department of Applied Biochemistry, Tokai University, Hiratsuka, Japan

**Keywords:** lamprey, agnathans, complement, C1q, variable lymphocyte receptors

## Abstract

Agnathans (lamprey and hagfish) are a group of primitive jawless fish. Jawed vertebrates possess adaptive immunity including immunoglobulins, while agnathans lack immunoglobulins but have alternative adaptive immunity in which variable lymphocyte receptors (VLRs) function as antibodies. The complement system consists of many proteins involved in the elimination of pathogens. In mammals, it is activated *via* the three different pathways, resulting in the generation of C3b followed by the lytic pathway. Complement components including C3, mannose-binding lectin (MBL), and MBL-associated serine proteases (MASP) of the lectin pathway and factor B of the alternative pathway have been identified from lamprey and/or hagfish, while lytic pathway components have not been identified. In mammals, C1q binds to IgM/IgG-antigen complexes and activates the classical pathway in association with C1r and C1s. Lamprey also has C1q (LC1q), but its function differs from that of mammalian C1q. LC1q acts as a lectin and activates C3 in association with MASP *via* the lectin pathway. Furthermore, LC1q may interact with a secreted type of VLR (VLRB) in complex with antigens and mediate activation of C3, potentially *via* MASP, leading to cytolysis. Cytolysis is mediated by a newly identified serum protein named lamprey pore-forming protein (LPFP). In conclusion, lamprey has a complement activation pathway, which could be regarded as the classical pathway and also has a cytolytic system that is distinct from the mammalian lytic pathway. Thus, it appears that the complement system of agnathans is very unique and may have developed independently from jawed vertebrates.

Lamprey and hagfish are among the extant species of primitive jawless fish (agnathans) that diverged from the ancestors of jawed vertebrates ~500 million years ago. Although it had generally been believed that agnathans have only innate immunity but lack adaptive immunity as is found in invertebrates, recent studies have shown that they possess an alternative adaptive immune system. In this system, variable lymphocyte receptors (VLRs), which are constructed of leucine-rich repeats similar to Toll-like receptors, are used to recognize foreign antigens (1, 2). VLR are functionally akin to the immunoglobulins in jawed vertebrates and are diversified by a gene conversion mechanism. Three *VLR* genes (*VLRA, VLRB*, and *VLRC*) have been identified in lampreys and hagfish. VLRA, VLRB, and VLRC are expressed on the surface of T-like lymphocytes, B-like lymphocytes, and a distinct lymphocyte lineage, respectively. In addition, VLRB is secreted as a multivalent protein and functions as a soluble antigen-specific “antibody” (3).

The complement system consists of many serum proteins involved in a chain reaction of proteolysis and protein complex assembly that leads to the elimination of pathogens. In mammals, the complement system is activated *via* three pathways, the lectin, classical, and alternative pathways (Figure 1A). The lectin pathway is activated by the binding of serum collagenous lectins, mannose-binding lectin (MBL), ficolin, and CL-LK [a heterocomplex of collectin liver 1 (CL-L1 alias collectin 10) and collectin kidney 1 (CL-K1 alias collectin 11)], to carbohydrates on the surfaces of pathogens. These lectins are complexed with MBL-associated serine proteases (MASPs). The classical pathway is activated by the binding of C1, the first complement component consisting of C1q, C1r, and C1s, to immune complexes. The alternative pathway is activated on the pathogen surface by the reaction of complement components including C3, factor B, and factor D. Complement activation *via* the three pathways results in the cleavage of C3 to generate C3b followed by C5 activation and C5b generation. C5b initiates the lytic pathway, in which C5b, C6, C7, C8, and C9 assemble, and polymerization of C9 generates a transmembrane pore. Complement components including C3, MBL, C1q, three types of MASP (MASP-1, MASP-A, MASP-B), factor B, short consensus repeatss (SCR)-containing control protein, and factor I have been identified from lamprey at the protein and/or DNA levels so far. It appears clear that agnathans have both the alternative and lectin pathways (4, 5). Late components (C5, C6, C7, C8, and C9) involved in the lytic pathway found in jawed vertebrates have not been identified in jawless fish. As will be described below, lamprey C1q was first discovered as a GlcNAc-binding lectin associated with MASP. This fact and the lack of immunoglobulins in jawless fish indicate that lamprey C1q is involved in the lectin pathway but not the classical pathway. As will also be described below, however, recent intriguing findings have shown that lamprey C1q binds to VLRB with concomitant activation of complement, which seems like the classical pathway of jawed vertebrates.

**Figure 1:**
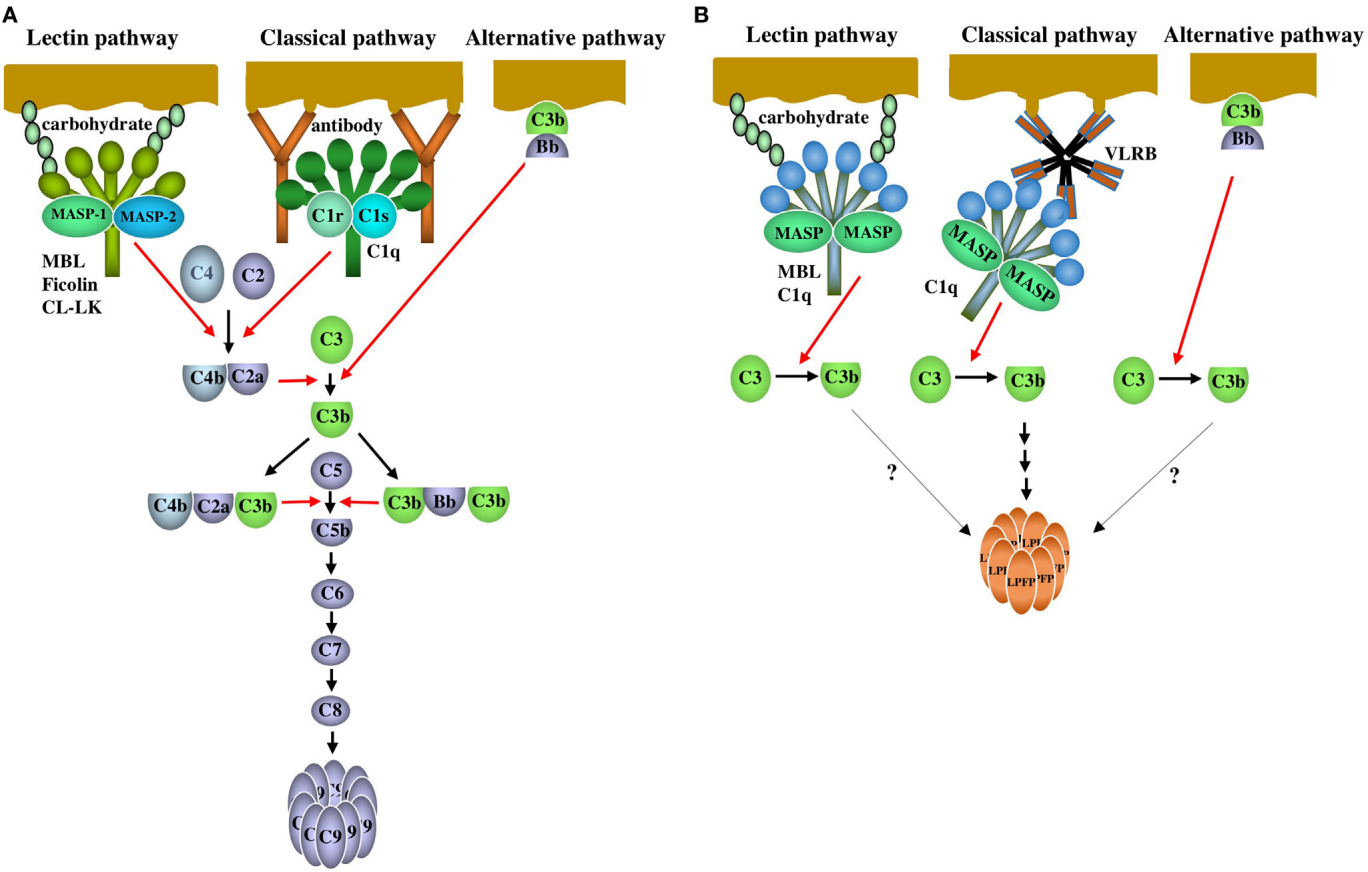
The complement system of mammals and agnathans. **(A)** Mammalian complement system. Lectin pathway: the lectin pathway is initiated by the binding of serum collagenous lectins [mannose-binding lectin (MBL), ficolin or CL-LK] to carbohydrates on pathogens. The lectins are complexed with MASP-1 and MASP-2. MASP-1 is auto-activated, and in turn, activates MASP-2. Activated MASP-2 cleaves C4 and C2 to generate a bimolecular C4bC2a complex. MASP-1 is also able to cleave C2. The C4bC2a complex acts as a C3 convertase that cleaves C3 into C3a and C3b. The trimolecular complex C4bC2aC3b acts as a C5 convertase cleaving C5 into C5a and C5b. Classical pathway: the classical pathway is initiated by the binding of C1 to antigen-antibody (IgG and IgM) complexes through C1q. C1r is auto-activated, and in turn activates C1s. Activated C1s cleaves C4 and C2 to generate a C3 convertase C4bC2a, followed by C3 and C5 activation as is the case with the lectin pathway. Alternative pathway: Factor B bound to C3b on the surface of pathogens is cleaved by factor D to generate Bb. The bimolecular C3bBb complex acts as a C3 convertase. C3b forms a trimolecular complex with C3bBb, which acts as a C5 convertase. After C5b is generated *via* the three activation pathways, C5b, C6, C7, C8, and C9 are assembled sequentially, leading to the polymerization of C9 (lytic pathway). **(B)** Agnathan complement system. Lectin pathway: MBL forms a complex with MBL-associated serine proteases (MASP) (MASP-1, MASP-A, and MASP-B). C1q forms a complex with MASP-A and possibly with MASP-1 and MASP-B. Upon binding of the MBL-MASP or C1q-MASP complexes to carbohydrates on pathogens, MASP changes from the unactivated form to the active form. As a result, activated MASP cleaves C3 into C3a and C3b. Which MASP is responsible for C3 cleavage is unknown. Classical pathway: VLRB, which forms a pentamer or tetramer of dimers, acts as an antibody and specifically binds to the antigens on pathogens. C3 is activated potentially *via* MASP associated with C1q that is bound to VLRB. Complement activation by C1q-MASP and VLRB leads to the polymerization of lamprey pore-forming protein (LPFP), resulting in the cytolysis of pathogens. Factors and mechanisms between the generation of C3b and the polymerization of LPFP are unknown. Alternative pathway: Although factor D has not been identified in agnathans, it is possible that C3 is activated by the C3bBb complex on pathogens as is the case with the mammalian alternative pathway. It remains unclear whether complement activation *via* the lectin and alternative pathways leads to LPFP-mediated cytotoxicity. Red and black arrows indicate proteolysis and reaction sequence, respectively.

Here, I review the complement system of agnathans, especially focusing on the lamprey C1q-mediated complement activation.

## C3

**Figure 2:**
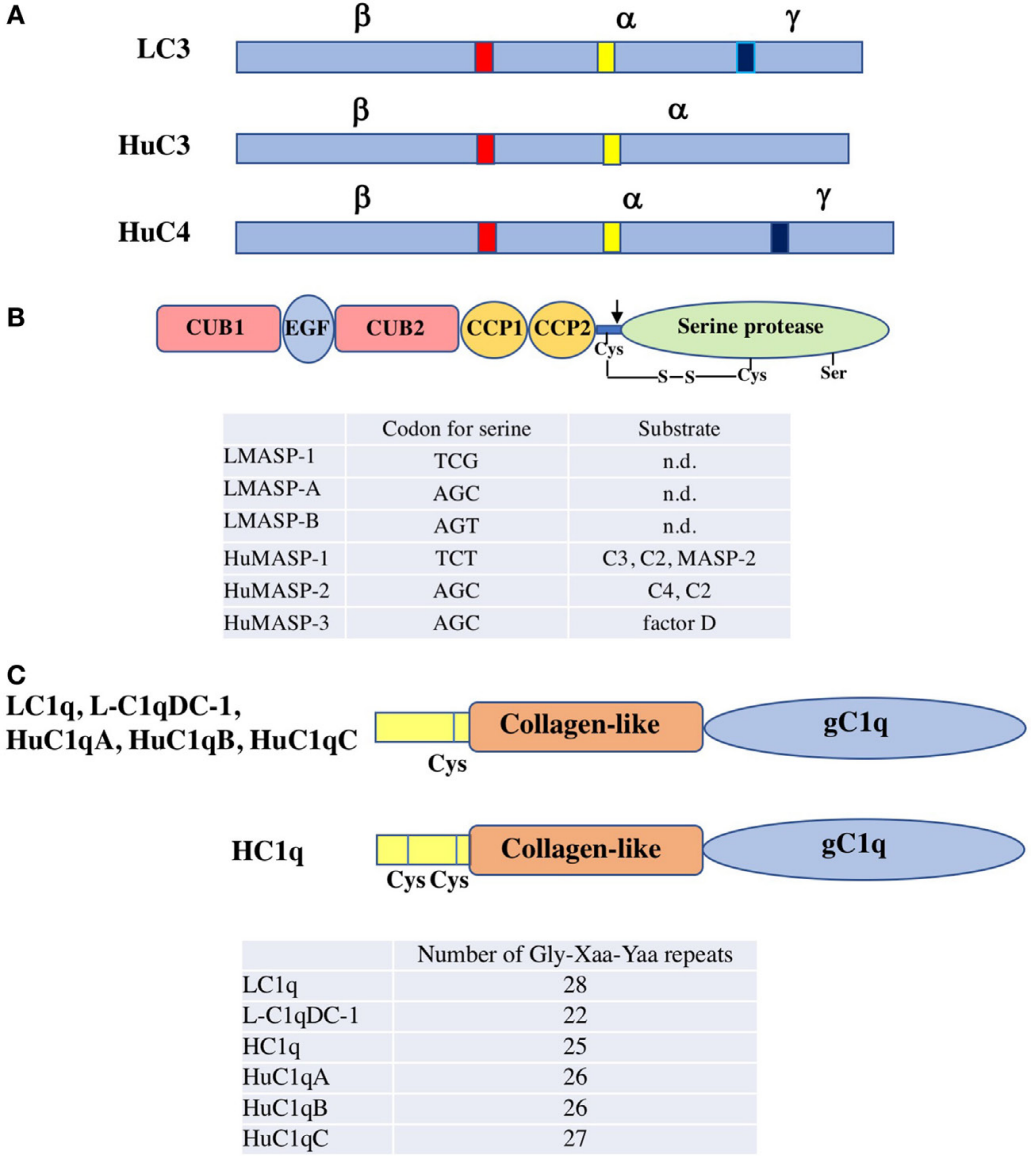
Structure of agnathan complement components. **(A)** Schematic representation of the primary structure of lamprey C3 (LC3), human C3 (HuC3), and human C4 (HuC4). The β − α and α − γ processing sites are shown in red and blue, respectively. These proteins are produced as a single polypeptide. After processing, the mature proteins with disulfide-bridged three polypeptides (α, β, and γ chains) or with disulfide-bridged two polypeptides (α and β chains) are produced. The thioester region is shown in yellow. **(B)** Domain structure of lamprey and human MASP. MASP consists of six domains, C1r/C1s/Uegf/bone morphogenetic protein (CUB) 1, epidermal growth factor, CUB 2, complement control protein (CCP) 1, CCP2 and serine protease domain. An unactivated form of MASP with a single polypeptide chain changes to an active form of MASP with two polypeptide chains linked by a disulfide bond by cleavage of a peptide bond (arrow). Codon for the serine residue essential for the catalytic activity in the protease domain of each MASP is listed. Substrates for each MASP are also summarized (n.d, not determined). LMASP-1, lamprey MASP-1; LMASP-A, lamprey MASP-A; LMASP-B, lamprey MASP-B; HuMASP-1, human MASP-1; HMASP-2, human MASP-2; HMASP-3, human MASP-3. **(C)** Domain structure of lamprey and human C1q. C1q consists of both collagen-like and gC1q domains. The number of Gly-Xaa-Yaa repeats of each C1q is listed. One cysteine residue is present in the N-terminal region for LC1q, L-C1qDC-1, HuC1qA, HuC1qB, and HuC1qC, while two cysteine residues for HC1q. HuC1qA, human C1qA chain; HuC1qB, human C1qB chain; HuC1qC, human C1qC chain.

Lamprey C3 isolated from lamprey *Lethenteron japonica* (*Lampetra camtschaticum*) serum has a molecular size of 190 kDa, consisting of three polypeptide chains (α, β, and γ) linked by disulfide bonds (6) (Figure 2A). This structural feature is in contrast to mammalian C3, which consists of two polypeptides linked by disulfide bonds. An internal thioester bond is present on the α chain of lamprey C3. Upon activation of the alternative complement pathway, C3b, a cleaved product of lamprey C3, covalently binds to substances such as zymosan and rabbit red cells (6) (Figure 1B). Lamprey C3 functions as an opsonin in phagocytosis of rabbit red cells by lamprey phagocytes. The cDNA cloning of lamprey C3 based on the amino acid sequences at the thioester region common to the mammalian C3, C4, and α_2_-macroglobulin has revealed that it has 31 and 22% identity with mouse C3 and C4 at the protein level, respectively (7). The possible β − α and α − γ processing sites are at the same positions as in mammalian C4, suggesting that lamprey C3 has three polypeptide chains as found in serum.

The hagfish (*Eptatretus burger*) C3 cDNA also has possible β − α and α − γ processing sites, but hagfish C3 isolated from serum consists of two chains, β and α + γ, suggesting incomplete processing of pro-C3 at the α − γ boundary in the hagfish (8–11).

The lamprey complement regulatory protein (Lacrep) is a serum protein consisting of eight SCRs (12). Lacrep is involved in the cleavage of C3b with a putative serum protease, thus regulating complement activation in the lamprey. Lamprey factor I could be one of candidates for the serum protease responsible for C3b cleavage (13).

## Factor B

Factor B (Bf) and C2 in mammals are the C3 cleaving enzymes of the alternative and classical pathways and share domain structure. Both have three SCR domains, a von Willebrand factor domain and a serine protease domain. Only a single Bf/C2 gene has been identified from lamprey and is located as an outlier in a phylogenetic tree of the jawed vertebrate Bfs and C2s (14). This suggests that the Bf/C2 gene duplication occurred in the jawed vertebrate lineage.

## MBL and MASP

Lamprey MBL is a homo-oligomer consisting of 25-kDa subunits, each of which is composed of 15 Gly-Xaa-Yaa repeats of the collagen-like domain in the N-terminal region followed by a carbohydrate-recognition domain (CRD) in the C-terminus (15). The message for MBL is expressed in the liver. Lamprey MBL binds to mannose, *N*-acetylglucosamine (GlcNAc) and glucose, but not to *N*-acetylgalactosamine (GalNAc) and galactose. Lamprey MBL is associated with three types of MASP (MASP-1, MASP-A, and MASP-B) in the serum. The serine residues essential for the catalytic activity in the protease domain of MASP-1 and MASP-A/MASP-B are coded by two distinct types, TCN type for the former and AGY type for the latter (16, 17) (Figure 2B). Lamprey MBL-MASP activates lamprey C3 to generate a band corresponding to the α’ chain in the fluid phase as assessed by SDS-PAGE, indicating the cleavage of C3 into C3a and C3b. Lamprey MBL-MASP also binds to yeast and activates C3 and the resulting C3b is deposited on the yeast surface. A question is raised as to which MASP is responsible for C3 activation. Lamprey MASP-1 and MASP-A/MASP-B are speculated to be orthologues of human MASP-1 and MASP-3, respectively, based on the codon usage for the serine residue in the serine protease domain as described above. Human MASP-1 is capable of cleaving C3 while MASP-2 and MASP-3 are not (18, 19). It is, therefore, possible that lamprey MASP-1 is involved in C3 activation in the MBL-MASP complex and that MASP-A and MASP-B have other functions such as the activation of pro-factor D of the alternative pathway (20). The MASP-1 cDNA with TCN type was identified from hagfish (21). As with MBL, CL-L1, and CL-K1 belong to the collectin family, whose members contain both a collagen-like domain and a CRD (22). In mammals, CL-LK, a heterocomplex of CL-L1 and CL-K1, forms complexes with MASPs and activates the lectin pathway as does MBL (23). Neither CL-L1 nor CL-K1 has yet been identified from agnathans.

## C1q and C1q-Mediated Complement Activation

Ficolins are a group of lectins having a collagen-like domain combined with a fibrinogen-like domain (24). Ficolins recognize the acetyl group moiety present in substances such as GlcNAc. In the course of a search for ficolins in the sera of lamprey using GlcNAc-agarose, we unexpectedly discovered C1q (LC1q) but not ficolins as a GlcNAc-binding lectin (25). Among the carbohydrates tested (GlcNAc, GalNAc, glucose, mannose, and galactose), LC1q exhibited its binding activity only toward GlcNAc. LC1q appears as 24 and 48 kDa protein bands under reducing and non-reducing conditions in SDS-PAGE, respectively. Its molecular size estimated by gel filtration is 480 kDa, indicating that LC1q is an oligomer consisting of identical 24-kDa polypeptides that form a dimeric structural unit linked by disulfide bond(s). The nine dimeric structural units (18 polypeptide chains) might interact non-covalently to give a hexameric structure, with each monomer consisting of three polypeptides similar to mammalian C1q, although mammalian C1q is composed of three distinct polypeptides (C1qA, C1qB, C1qC). Each subunit of LC1q is composed of a collagen-like domain with 28 Gly-Xaa-Yaa repeats and a C-terminal globular C1q (gC1q) domain (Figure 2C). LC1q has one cysteine residue in the N-terminal region, which might be involved in formation of a dimeric structural unit. The binding site for GlcNAc is thought to be located in the gC1q domain. LC1q shares ~35% identity at the amino acid level with mammalian C1q. Mammalian C1q is associated with C1r and C1s and binds to immunoglobulins (IgG and IgM) with concomitant activation of C4 and C2 *via* the classical pathway. LC1q is complexed with MASP-A in a Ca^2+^-dependent manner (25) and possibly with MASP-1 and MASP-B as well. LC1q-MASP cleaved C3 to generate C3b in the fluid phase as is seen in lamprey MBL-MASP. These results suggest that C1q may have emerged as a recognition lectin in innate immunity before the establishment of immunoglobulins in the cartilaginous fish and that in association with MASP, it activates complement *via* the lectin pathway to generate C3b upon binding to carbohydrates on the surfaces of microbes (Figure 1B). Mammalian C1q binds not only immunoglobulins but also to microbial components such as lipoteichoic acid with concomitant activation of complement, suggesting that it still has a role in innate immunity (26). From an evolutional point of view, it is of interest that ficolins have not been identified from agnathans (lamprey), chondrichthyans (hammerhead shark), or osteichthyans (27, 28), although they are found in invertebrates (4). It appears that C1q acts as a GlcNAc-binding lectin in the lectin pathway instead of ficolins as far as agnathans are concerned. Another type of C1q-like protein (L-C1qDC-1) is present in lamprey serum (29). It remains unknown whether L-C1qDC-1 has a lectin activity and forms complexes with MASP as does LC1q.

Yamaguchi et al. reported the isolation of C1q (HC1q) from the sera of hagfish (30). GlcNAc-agarose was used for its isolation as was the case with LC1q. Interestingly, HC1q was not eluted from GlcNAc-agarose with GlcNAc but with EDTA. HC1q was demonstrated to bind directly to Sepharose in a Ca^2+^-dependent manner, indicating its binding to agarobiose that is a constituent carbohydrate of the matrix of GlcNAc-agarose. Unlike LC1q, HC1q exhibits a mixture of homopolymers appearing as approximately 50, 105, 165, and 250 kDa protein bands in SDS-PAGE. HC1q homopolymer consists of 26-kDa polypeptides, each of which is composed of an N-terminal region containing two cysteine residues, a collagen-like domain with 25 Gly-Xaa-Yaa repeats followed by a gC1q domain (Figure 2C). LC1q and HC1q show ~35% identity at the amino acid level. The multiple homopolymers of HC1q could be ascribed to disulfide bonds among the polypeptides with two cysteine residues in the N-terminal region. It remains unknown whether HC1q is associated with MASP. HC1q binds to various bacteria, fungi and their components including *E. coli, S. aureus, S. cerevisiae*, and peptidoglycan in a Ca^2+^-dependent manner.

In 2013, Wu et al. reported that the antisera of lamprey exhibit cytotoxicity against the corresponding antigens including rabbit erythrocytes, *E. coli* and Hela cells (31). By depletion experiments, VLRB, LC1q, and C3 in the antisera were demonstrated to be essential for cytotoxicity. Furthermore, adding back the VLRB to the VLRB-depleted antisera restored cytotoxicity. VLRB and LC1q were deposited on the surface of Hela cells treated with anti-Hela serum. Co-precipitation experiments using a combination of immunoprecipitation and Western blotting showed that VLRB was associated with LC1q in antisera as well as in non-immune sera. During the cytolytic process, C3b generated as a result of C3 activation was deposited on the surface of target cells. Although it was not examined in this study whether LC1q was associated with MASP, it is possible that MASP associated with LC1q was responsible for the C3 activation. L-C1qDC-1, another type of lamprey C1q, was also demonstrated to be associated with VLRB in non-immune serum (29). In addition, L-C1qDC-1 and VLRB were found to colocalize on the surface and in the cytoplasm of Hela cells and MCF-7 cells after incubation of these cells with serum. It remains unknown whether L-C1qDC-1 activates C3 as does LC1q.

## Lamprey Pore-Forming Protein (LPFP)

A serum protein termed LPFP has been demonstrated to be involved in VLRB-mediated complement cytotoxicity (32). LPFP was identified as a protein deposited on the membrane of *E. coli* treated with lamprey anti-*E. coli* sera. cDNA cloning has revealed that LPFP consists of a jacalin-like lectin domain and an aerolysin-like domain. Jacalins are a group of lectin that was originally isolated from jackfruit (*Artocarpus heterophyllus*) seeds and they are divided into two groups depending on the carbohydrate specificity, galactose-specific and mannose-specific lectins (33). Aerolysin is a pore-forming toxin produced by *Aeromonas* species and binds to eukaryotic cell membrane leading to pore formation (34). Many proteins have been reported to have an aerolysin-like domain (35, 36). Dln1 is a zebrafish protein with both a jacalin-like lectin domain and an aerolysin-like domain like LPFP. Dln1 and LPFP show 56.5% identity. Dln1 binds preferentially to high-mannose glycans. It has recently been proposed for the pore-formation of Dln1 that it forms an antiparallel dimer in which one monomer binds to glycosylated extracellular receptors on the target cells through its jacalin-like lectin domain and subsequently dissociate into transient two parallel monomers, both of which binds to receptors (37). The Dln1 monomers then oligomerize to form an octameric pre-pore, leading to a transmembrane octameric pore in which the aerolysin-like domain penetrates the membranes. The oligomerization of Dln1 is accelerated at the microenvironments of relative low pH. LPFP binds to yeast mannan and polymerizes like Dln1. The hub-and-spoke wheel structure was observed on the membrane of cells treated with antisera, which can be ascribed to LPFP polymerization as is the case with Dln1. The function of LPFP appears to be equivalent to C9 of the late components in mammals. The entire process of the VLR-mediated cytotoxicity remains unknown. It is, however, most likely that VLRB acting as an antibody binds to microbes (antigens), followed by C3 activation by LC1q-MASP bound to VLRB. This activation process is in parallel with the classical pathway in mammals. In this case, it is unknown whether the binding between LC1q and VLRB are mediated by lectin-carbohydrate interactions, and also factors and mechanisms involved in the process between C3 activation and LPFP polymerization remains to be elucidated.

## Conclusion

LC1q has two distinct roles in complement activation in the lamprey. First, LC1q directly binds to microbes *via* its lectin activity and activates C3 by the associated MASP *via* the lectin pathway. In this case, LC1q can be regarded as the prototype of jawed vertebrate C1q before the emergence of immunoglobulins in Chondrichthyes. Second, LC1q and VLRB form a complex. Upon binding of VLRB to microbial antigens, LC1q activates C3 potentially by MASP associated with LC1q, resulting in LPFP-mediated cytolysis of the antigen (Figure 1B). In this case, the reaction sequence of LC1q, VLRB, and microbial antigens remains to be elucidated. The presence of the LCq-VLRB complexes in lamprey sera strongly suggests that the preformed complexes bind to antigens that VLRB recognize, followed by complement activation. This is in contrast to the mammalian classical pathway in which antibodies first bind to antigens and then C1 binds to the antigen–antibody complexes. It remains unknown whether a similar reaction sequence to the mammalian classical pathway also takes place in the agnathans.

In conclusion, agnathans have a complement activation pathway, which could be regarded as the classical pathway and also has a cytolytic system that is distinct from the mammalian lytic pathway. This mechanism of complement system is very unique and has not been reported for vertebrates other than agnathans so far. Further studies are expected to determine whether C3 activation *via* the lectin pathway involving LC1q and MBL and *via* the alternative pathway leads to the LPFP-mediated cytotoxicity as is the case with VLR-mediated cytotoxicity. It appears that the complement system of agnathans differs from that of jawed vertebrates and may, at least in part, have evolved independently.

## Author Contributions

The author confirms being the sole contributor of this work and approved it for publication.

## Conflict of Interest Statement

The author declares that the research was conducted in the absence of any commercial or financial relationships that could be construed as a potential conflict of interest.
